# Short Forms of the Cross-Cultural (Chinese) Personality Assessment Inventory: Reliability, Validity, and Measurement Invariance Across Gender

**DOI:** 10.3389/fpsyg.2021.709032

**Published:** 2021-12-15

**Authors:** Mingjie Zhou, Duan Huang, Fen Ren, Weiqiao Fan, Weiqi Mu, Fugui Li, Jianxin Zhang, Fanny M. Cheung

**Affiliations:** ^1^Cas Key Laboratory of Mental Health, Institute of Psychology, Chinese Academy of Sciences, Beijing, China; ^2^Department of Psychology, University of Chinese Academy of Sciences, Beijing, China; ^3^Department of Psychology, Wuhan Sports University, Wuhan, China; ^4^Hubei Key Laboratory of Exercise Training and Monitoring, Wuhan Sports University, Wuhan, China; ^5^School of Education and Psychology, University of Jinan, Jinan, Shandong, China; ^6^Department of Psychology, Shanghai Normal University, Shanghai, China; ^7^Department of Psychology, Chinese University of Hong Kong, Shatin, China

**Keywords:** CPAI, CPAI-2, interpersonal relatedness, social potency, ESEM, measurement invariance

## Abstract

Filling out long questionnaires can be frustrating, unpleasant, and discouraging for respondents to continue. This is why shorter forms of long instruments are preferred, especially when they have comparable reliability and validity. In present study, two short forms of the Cross-cultural (Chinese) Personality Assessment Inventory (CPAI-2) were developed and validated. The items of the short forms were all selected from the 28 personality scales of the CPAI-2 based on the norm sample. Based on some priori criteria, we obtained the appropriate items and constructed the 56-item Chinese Personality Assessment Inventory (CPAI) and the 28-item CPAI. Then, we examined the factor structure of both short forms with Exploratory SEM (ESEM) and replicated the four-factor structure of the original CPAI-2, reflecting the four personality domains of Chinese people, namely, Social Potency, Dependability, Accommodation, and Interpersonal Relatedness. Further analyses with ESEM models demonstrate full measurement invariance across gender for both short forms. The results show that females score lower than males on Social Potency. In addition, these four factors of both short forms have adequate internal consistency, and the correlation patterns of the four factors, the big five personality traits, and several health-related variables are extremely similar across the two short forms, reflecting adequate and comparable criterion validity, convergent validity, and discriminant validity. Overall, the short versions of CPAI-2 are psychometrically acceptable and have practically implications for measuring Chinese personality and cross-cultural research.

## Introduction

Lengthy, time-consuming questionnaires may evoke impatience or frustration in respondents, leading to temporary measurement errors and increasing the likelihood of careless responses, withdrawal from data collection, and refusal to further participation (Schmidt et al., 2003; Donnellan et al., 2006). Consequently, brief measures within the framework of the big five model have become increasingly available and shorter, including the 60-item NEO five-factor inventory (NEO-FFI, Costa and McCrae, 1992), the 44-item Big Five Inventory (BFI, John et al., 2008), the 30-item BFI-2-S and 15-item BFI-2-XS (Soto and John, 2017a), the 20-item Mini International Personality Item Pool (Donnellan et al., 2006), and even the 10-item short version of BFI (Rammstedt and John, 2007). These widely used measures have demonstrated that the short version is sufficient to provide a valuable assessment of personality constructs (e.g., Dale et al., 2020; Perry et al., 2020; Shchebetenko et al., 2020).

However, the big five model has been challenged in terms of cross-cultural adaptability (Cheung et al., 2011; Li et al., 2019; Wang et al., 2019; Dong et al., 2021). As a theory derived in western society, the big five model may include specific traits that are more valued in western societies than in non-western societies (Church, 2001), or it may not include some traits that are more prominent in non-western societies than in western societies. To avoid these blind spots, Cheung et al. (2011) proposed the combined etic-emic approach that can take into account both cultural-specific (indigenous) and cultural-universal personality traits. Using this approach, several forms are developed, namely, the Chinese Personality Assessment Inventory (CPAI, Cheung et al., 1996), the Cross-Cultural (Chinese) Personality Assessment Inventory (CPAI-2, Cheung et al., 2008), and the Cross-cultural (Chinese) Personality Assessment Inventory for Adolescents (CPAI-A, Cheung et al., 2008).

The CPAI measures can serve as omnibus indigenous personality inventories for the Chinese people and as cross-culturally valid instruments for people from non-Chinese societies (Cheung et al., 2003; Wada et al., 2004; Born and Jooren, 2009; Iliescu and Ion, 2009; Dang et al., 2010; Cheung et al., 2013). However, there are too few short forms of CPAI measures compared to the prosperity of the brief measures of the big five model, and even only one short form of the CPAI-A has been developed recently (Dong et al., 2021). In the present study, we developed two short forms for the CPAI-2.

To develop the CPAI, researchers explored multiple sources of folk personality descriptions, including contemporary Chinese novels, Chinese proverbs, and psychological research literature. They collected descriptions about oneself from an informal street survey and descriptions about others from surveys of various professionals (Cheung et al., 1996). At the same time, the researchers drew on the existing Western personality measurement literature. The CPAI personality profile were generated from those descriptions with an integrated and balanced treatment of universal and culture-specific aspects, including 22 normal personality scales, 12 clinical scales, and 3 validity scales with a total of 510 items. To date, the CPAI has been developed and repeatedly revised over 20years, resulting in two versions: an adolescent version (CPAI-A) and an adult version (CPAI-2). The adult version, CPAI-2, consists of 28 normal personality scales, 12 clinical scales, and 3 validity scales with a total of 541 items. The present study focuses on the normal personality scales (Form B).

Explanatory factor analyses reveal that the 28 personality scales of the CPAI-2 reflect four deeper latent domains, namely, Social Potency, Dependability, Accommodation, and Interpersonal Relatedness (IR; Cheung et al., 2008), which are identical to the structure of the original CPAI personality scales. Of particular note is that the IR factor contains more indigenous elements in Chinese culture, such as paying attention to reciprocity in the relationship, avoiding face-to-face conflict, maintaining superficial harmony, and saving face for everyone, which highlights the attitudes, beliefs, and behavioral patterns of how Chinese people “behave” in instrumental interpersonal relationships. In a joint factor analysis of the CPAI and the NEO PI-R, IR did not load on any of the NEO PI-R factors (Cheung et al., 2001). In another joint analysis of the CPAI-2 and the NEO-FFI, IR was again distinct (Cheung et al., 2008). That is to say, IR is juxtaposed with the five personality traits defined in the big five model, resulting in a “big six” personality structure. At the same time, Social Potency, Dependability, and Accommodation were intertwined with the big five personality traits in these joint factor analyses, showing more cultural-universal characteristics.

The four-factor structure of the CPAI and CPAI-2 has been replicated in several English-speaking groups, including Singapore Chinese adults and Caucasian American college students (Cheung et al., 2003), Chinese Americans and European Americans (Lin and Church, 2004), and a mixed Singapore sample including Chinese, Malays, and Indians (Cheung et al., 2006). Similarly, the big six personality structure has been found in English-speaking groups, including Hawaiian Students and Chinese Singaporeans through joint factor analysis (Cheung et al., 2001, 2003). These findings suggest that the IR factor may also be present in the personality structure of Westerners. To date, CPAI-2 has been translated into five languages other than English, including Japanese (Wada et al., 2004), Korean (see Cheung et al., 2013), Vietnamese (Dang et al., 2010), Dutch (Born and Jooren, 2009), and Romanian (Iliescu and Ion, 2009). Factor analysis of these translations showed that IR can still be established independently. These findings prompted researchers to consider the cross-cultural validity of the CPAI-2 and to rename it the Cross-cultural (Chinese) Personality Assessment Inventory.

In addition to the structural cross-cultural comparisons, comparisons of group means revealed significant differences across cultures and genders (Cheung et al., 2004; Lin and Church, 2004). One study reported cultural mean differences on the CPAI-2, with less acculturated Asian Americans scoring higher on the IR compared to more acculturated Asian American and European American participants (Lin and Church, 2004). Another study reported gender differences, with males scoring higher on most scales of the Social Potency factor and some scales of the Dependability factor and females scoring higher on some scales of the Dependability factor, Accommodation factor, and Interpersonal Relatedness factor (Cheung et al., 2004). We can improve the comparison of group means on the CPAI measures by addressing the following two issues. Firstly, Domain-level gender differences of CPAI-2 remained unrevealed. Secondly, all these mean score comparisons were conducted without establishing measurement invariance (MI) across groups, which results in mean differences that cannot be directly explained (Cheung and Rensvold, 2002).

For more than two decades, a series of studies have been conducted with the CPAI-2, highlighting its value in predicting important aspects of people’s lives, including adolescent life satisfaction (Ho et al., 2008; Xie et al., 2016), adolescent loneliness (Li et al., 2019), career exploration of university students (Fan et al., 2012), personal decision-making style (Gan et al., 2019), urban entrepreneurial dynamism (Obschonka et al., 2019), and so on. In these studies, indigenous personality traits, such as IR, demonstrated additional predictive power. More empirical studies are needed to examine the role of CPAI-2 in understanding and predicting human behavior cross cultures.

The 28 personality scales of CPAI-2 have a total of 298 items that takes about half an hour to finish, a time long enough to provoke impatience and eliminate the capacity of other variables, limiting the application of the CPAI-2. Thus, the present study aimed to develop two short forms for the CPAI-2: the 56-item CPAI and the 28-item CPAI. The former took two items from each of the 28 personality scales, with the aim of reducing the number of items and retaining a certain degree of hierarchical measurement suitable for both domain-level measurement and scale-level measurement. The latter removes one of the two items and saves more time, though it suffers from the loss of hierarchical measurement. That is, the former retains a certain degree of hierarchical measurement, while the latter is more time efficient and suitable for studies where time of assessment and respondent fatigue are the core questions. Table 1 demonstrates the item numbers of each scale for the original CPAI-2, the 56-item CPAI and the 28-item CPAI.

**Table 1:** Item numbers of each of the 28 personality scales for the original CPAI-2, the 56-item CPAI and the 28-item CPAI, respectively.

Domains	Scales	Item Numbers		
		CPAI-2	56-CPAI	28-CPAI
SP	Novelty	10	2	1
	Diversity	10	2	1
	Divergent Thinking	10	2	1
	Leadership	10	2	1
	Logical vs. Affective Orientation	10	2	1
	Aesthetics	10	2	1
	Extraversion vs. Introversion	10	2	1
	Enterprise	10	2	1
De	Responsibility	10	2	1
	Emotionality	10	2	1
	Inferiority vs. Self-Acceptance	18	2	1
	Practical Mindedness	12	2	1
	Optimism vs. Pessimism	10	2	1
	Meticulousness	10	2	1
	Face	11	2	1
	Internal vs. External Locus of Control	10	2	1
	Family Orientation	10	2	1
Ac	Defensiveness (Ah-Q Mentality)	10	2	1
	Graciousness vs. Meanness	10	2	1
	Interpersonal Tolerance	10	2	1
	Self vs. Social Orientation	10	2	1
	Veraciousness vs. Slickness	10	2	1
IR	Traditionalism vs. Modernity	15	2	1
	Ren Qing (Relationship Orientation)	12	2	1
	Social Sensitivity	10	2	1
	Discipline	10	2	1
	Harmony	12	2	1
	Thrift vs. Extravagance	8	2	1
Total number of items		298	56	28

*SP, Social Potency; De, Dependability; Ac, Accommodation; IR, Interpersonal Relatedness*.

When developing the 56-item CPAI and the 28-item CPAI, we tried to make both short forms retain the same hierarchical structure as the original CPAI-2 and maintain adequate reliability and validity. As for the structure, we wanted the short forms to reflect the four distinct domains, each with the same content bandwidth as the CPAI-2. The way we selected items ensured that the short forms would completely cover the content of the CPAI-2 and retain the original structure at the scale-level. To obtain adequate reliability and validity, we used a combination of empirical and rational criteria. Empirically, authors familiar with the CPAI-2 were responsible for item selection based on their conceptual judgment regarding the extent to which the content of the selected items represented the overall meaning of their underlying traits. Rationally, we tended to select or retain items with higher factor loadings, less cross-loading problems and items that contribute to higher alpha coefficients for domains. More importantly, we wanted to demonstrate that the short forms did perform well in terms of these psychometric qualities. In general, we had three goals in present study.

**Figure 1:**
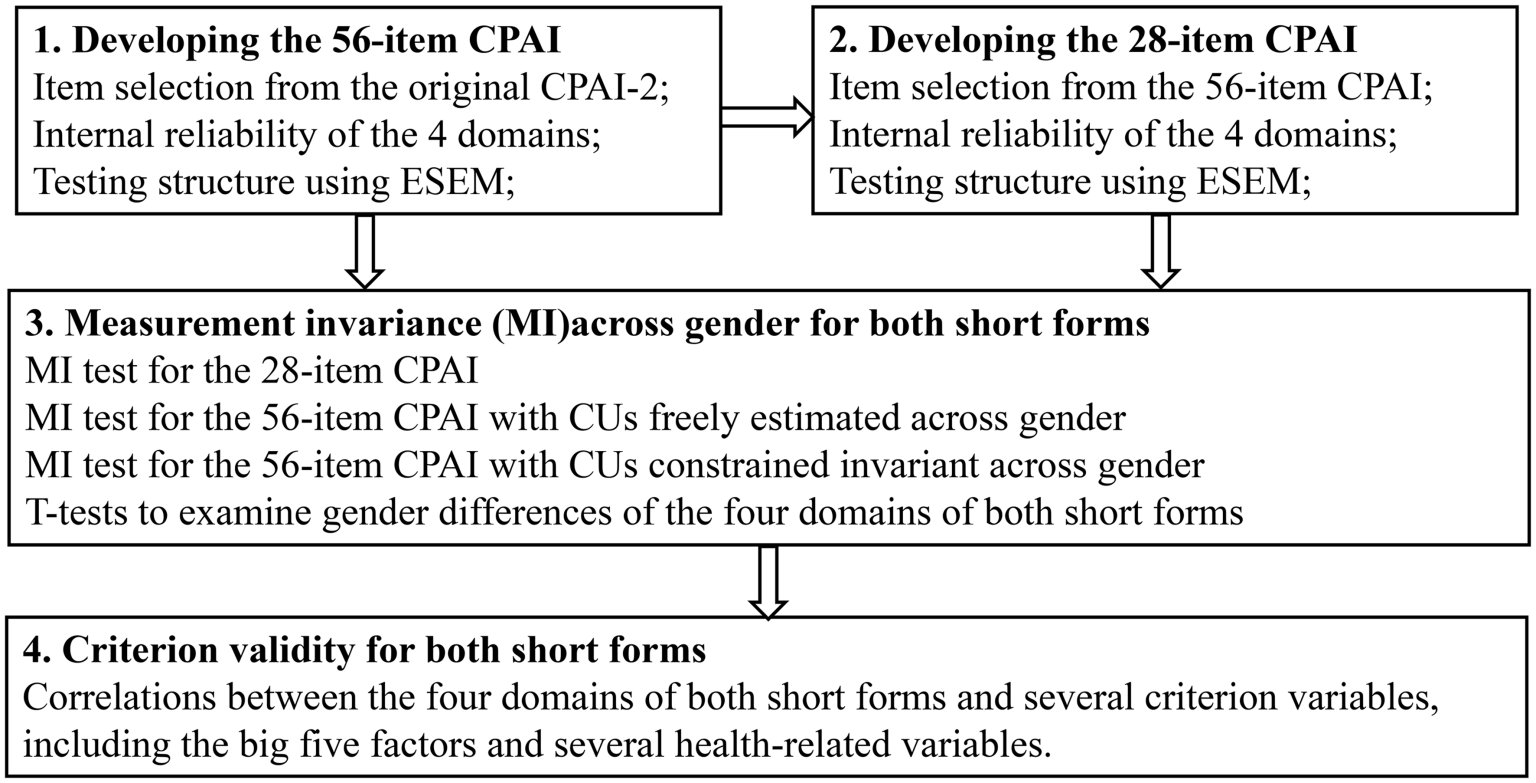
Workflow of this study.

Firstly, as mentioned above, we selected items from the 28 personalities scales of the CPAI-2 for the two short forms based on some priori criteria. Secondly, we investigated the four-factor structure of the short forms and tested their measurement invariance across gender. Finally, we tested the criterion validity of the short forms by examining the relationship between the factors of the short forms and several important variables. Figure 1 demonstrated the workflow of this study.

## Materials and Methods

### Participants

The study analyzed data from 2 samples: one for item selection and construct validity and one for criterion validity. A paper-pencil measurement was administered to 11,492 Chinese residents for item selection and construct validity. Of those, 355 submitted incomplete questionnaires. Thus, 11,137 Chinese residents (50.0% female, 49.1% male, 0.9% not reporting gender) provided complete data that were included for statistical analyses. Their median age was 40, and 96% were between 18 and 72years old. They came from 7 provinces, including Fujian (10.4%), Henan (25.7%), Liaoning (10.4%), Qinghai (4.3%), Sichuan (20.8%), Shandong (18.8%), and Zhejiang (9.7%). To examine the criterion validity of the short forms, data collection was conducted online. 330 participants (69.4% female, 30.6% male) completed the questionnaire. Their age ranges from 18 and 59years (.=26.11, *SD*=7.22). Of these, 61.2% were students, and 87.2% had an undergraduate or graduate degree. Note that there was no intersection between the two samples.

### Measures

Questionnaire instruments include the CPAI-2, the Ten Item Personality Inventory (TIPI, Gosling et al., 2003), the Patient Health Questionnaire (PHQ-9, Kroenke and Spitzer, 2002), the Generalized Anxiety Disorder Screener (GAD-2, Kroenke et al., 2007), the General Health Questionnaire (GHQ-12, Goldberg and Williams, 1988), and the Subjective Well-Being Scale (Andrews and Withey, 1976).

#### CPAI-2

We used the traditional Chinese version of the CPAI-2 in this study. The original CPAI-2 uses a true-false rating scale, while the two short versions use a 5-point Likert scale. That is, respondents were asked to rate each statement depicting personal characteristics or typical behaviors describing their personality, from 1 (least) to 5 (most).

#### TIPI-10

The TIPI-10 is a self-rated questionnaire containing 10 items, each on a 7-point Likert scale (1=disagree strongly, 7=agree strongly). A study showed that TIPI-10 can used as a reliable and effective instrument to measure the Big Five Personality in a Chinese sample (Li, 2013).

#### Patient Health Questionnaire

The PHQ-9 was used to assess the severity of depressive symptoms over a two-week period. The scale includes nine items on a 4-point Likert scale (1=not at all, 4=nearly every day). The higher the total score, the more severe the depressive symptoms. A previous study indicated that PHQ-9 has good psychometric qualities in Chinese samples (Wang et al., 2014). Cronbach’s α for the PHQ-9 in this study was 0.906.

#### Generalized Anxiety Disorder Screener

The GAD-2 consists of two core criteria for generalized anxiety disorder. The scale uses a 4-point Likert scale ranging from 1 (not at all) to 4 (nearly every day). The higher the total score, the more severe the generalized anxiety disorder. Cronbach’s α in this study was 0.840.

#### General Health Questionnaire

The GHQ comprises 12 items with a 4-point response scale ranging from “rarely” to “almost always.” The total score was used to indicate the severity of mental health problem. The higher the total score, the more serious the mental health problem. Cronbach’s α was 0.898 for the GHQ in the present study. In addition, the GHQ consists of three sub-dimensions: social dysfunction, anxiety, and loss of confidence (Graetz, 1991). Cronbach’s *α* was 0.878, 0.786, and 0.856 for the three sub-dimensions, respectively.

#### Subjective Well-Being Scale

The scale has only one question and comprises seven faces, ranging from 1 (very happy) to 7 (very sad). Specifically, participants should determine which face is closest to their overall life experience and select the appropriate option. The happier the picture the participant chose, the higher their overall level of subjective well-being.

### Data Analysis

The analyses were performed with Mplus 8.4 (Muthén and Muthén, 1998–2017) and SPSS20.0 software. Mplus 8.4 was used to test the structure and measurement invariance of the short forms, while SPSS20.0 was used to calculate the alpha coefficients, conduct t tests, and test criterion validity.

We mainly used Exploratory SEM (ESEM) rather than CFA to explore factor structure, correlations among factors and measurement invariance across gender for the two short forms. The CFA models require that cross-loadings of items be set to zero, a limitation that may lead to two problems (Asparouhov and Muthen, 2009; Marsh et al., 2014). Firstly, it is almost impossible for item-level CFAs to get an acceptable fit (e.g., CFI, TLI>0.9; RMSEA<0.05) for instruments that are well established in EFA research. Secondly, the factor correlations in CFA are likely to be positively biased, sometimes substantially so. Marsh et al. (2014) regarded ESEM as an overarching integration of the best aspects of CFA and EFA, since ESEM can perform almost all the functions of CFA and is immune to both problems. Previous studies on the BFI and other FFA measures have demonstrated that, compared with CFA models, ESEM models have a better fit, smaller factor correlations, and almost identical factor loadings (Marsh et al., 2010; Chiorri et al., 2016).

The analysis of ESEM used a robust maximum likelihood estimator with standard errors and fit tests that were robust concerning the non-normality of the observations (Muthén and Muthén, 1998–2017). As done by Marsh et al. (2010), we used an oblique GEOMIN rotation (the default in Mplus) in ESEM. Related material is available at the Open Science Framework https://mfr.osf.io/render?url=https%3A%2F%2Fosf.io%2Fg359z%2Fdownload.

### Correlated Uniquenesses

Following Chiorri et al. (2016), we included ESEM models with and without *a priori* correlated uniquenesses (CUs; covariances between specific variance components associated with two different items of the same CPAI scale).

The model fit can be improved by freeing the correlations among error covariances (CUs) of some items, a strategy that is legitimate only in limited case that these items have further common variance beyond that explained by the specified latent factors (Marsh et al., 2010). The common variance beyond those caused by a common factor may result from a common method (e.g., Marsh et al., 1992), similar item wording (e.g., Chiorri et al., 2016) or “specific” factors that are independent of the “general” factor (e.g., Marsh et al., 2010, 2013; Chiorri et al., 2016). Marsh et al. (2010) posited that items from the same facet of a specific Big Five factor have higher correlations than items from different facets of the same Big Five factor. They claimed that inflated correlations could be divided into those could be explained in terms of the common Big Five factor and those could track back to the same facet and suggested modeling the correlations due to facets as CUs by freeing the correlations among error covariances of each pair of items from the same facet, an approach that always leads to a considerable increase in model fit (Marsh et al., 2013; Chiorri et al., 2016).

The four deep domains of the CPAI-2 consist of 28 personality scales, of which 8 scales are Social Potency, 9 scales are Dependability, five scales are Accommodation, and six scales are Interpersonal Relatedness (see Table 1). We selected the same number of items from each scale to construct the short forms of the CPAI in an attempt to maintain the hierarchical structure of the original CPAI and to avoid the “bandwidth-fidelity dilemma” (Cronbach and Gleser, 1957). Thus, there are 28 pairs of items in the 56 item CPAI, and each pair comes from the same scale, in which case *a priori* set of 28 CUs should be included in the four-factor model of the 56-item short form to cope with correlation inflation due to shared scales. We also set another CU to attain an adequate model fit, a CU due to a wording effect rather than from the same scale. That is, we specified *a priori* set of 29 CUs in total.

### Measurement Invariance Models

Marsh et al. (2014) recommended a 13-model taxonomy of invariance tests that can be conducted within an ESEM framework. According to the 13 models, we applied increasingly stringent equality constraints on the measurement parameters between male and female participants.

Four particularly noteworthy levels of invariance, from least to most strict, were configural, weak, strong, and strict invariance (Meredith, 1993). Configural invariance specifies the same number of factors with same items across groups and does not require any estimated parameters to be the same. It serves as a baseline for comparing other models that impose equality constraints on the parameters across groups. The ability of the configural invariance model to fit the data must be tested. The weak invariance model requires that factor loadings to be invariant across groups. Strong invariance model constrains both factor loading and intercepts (indicator means) to be equal across groups. If the strong invariance model is supported, the changes in the latent factor means can be reasonably interpreted as changes in the latent constructs. However, strong invariance is a necessary, but not sufficient, condition for testing manifest group mean differences. The differences in item reliability across groups will distort the observed mean differences in scores. The strict invariance model is sufficient because it adds a constraint of invariant residual variances (item uniquenesses) to strong invariance, indicating that item reliability is invariant.

The taxonomy of 13 models also includes invariance of the latent means and of the factor variance-covariance matrix. The former assumes at least strong invariance and sets the factor means to zero in both groups, while the latter assumes at least weak invariance and adds constraints on invariant factor variances and covariances.

### Goodness of Fit

Marsh et al. (2010) recommend the following fit indices independent of sample size: the comparative fit index (CFI), the Tucker-Lewis index (TLI), the root mean square error of approximation (RMSEA), and the significance of parameter estimates. We also reported the robust chi-square test statistic, a fit index very sensitive to sample size. For the RMSEA, values less than 0.08 and 0.05 are considered as acceptable and optimal fits, respectively. For the CFI and TLI, values greater than 0.90 and 0.95 are considered as acceptable fits and optimal fits, respectively (Marsh et al., 2004).

We used the change in CFI (ΔCFI) and the change in RMSEA (ΔRMSEA) to compare the relative fit of two nested invariance models. ΔCFI less than 0.01 or/and ΔRMSEA less than 0.015 supports a more parsimonious model and provides evidence of invariance at the given level (Cheung and Rensvold, 2002; Chen, 2007). In addition, if TLI or RMSEA is as good as or better than the more complex model, the more parsimonious model is supported, which is a relatively conservative guideline (Marsh, 2007).

## Results

### Developing the 56-Item CPAI

We created a 56-item CPAI by selecting two items from each of the 28 personality scales of CPAI-2. The item selection process has two stages. The first stage applied empirical criteria, while the second stage applied rational criteria. In the first stage, two authors independently selected two items from each of the 28 personality scales based on their own conceptual judgments regarding the extent to which the content of the items represents their underlying traits. If they selected different items from the same scale and could not come to an agreement, then all selected items were retained for screening at the next stage. In this stage, we got 68 items with 9 scales having more than 2 items because of disagreement, including Internal vs. External Locus of Control (I_E, 4 items), Responsibility (Res, 3 items), Self vs. Social Orientation (S_S, 3 items), Traditionalism vs. Modernity (T_M, 3 items), Ren Qing (Ren, 4 items), Social Sensitivity (Soc, 3 items), Discipline (Dis, 3 items), Harmony (Har, 4 items), and Thrift vs. Extravagance (T_E, 3 items).

In the second stage, we collected data of the 68 items on 5-point Likert scale and used domain-level alpha coefficient as criteria to reduce items. For Social Potency, items reduction was not necessary because none of the 8 scales had more than 2 items. For Dependability, 2 of the 9 scales had more than 2 items, that is, I_E and Res had 4 and 3 items, respectively. Then, there were 6 possible solutions for selecting two items from I_E and 3 from Res. We combined each of the 4 items selected from I_E and Res with items from other scales of Dependability and got 18 possible combinations (6*3) in total. The alpha coefficients of these combinations were from 0.821 to 0.831, with an average of 0.826. Finally, we chose the combination with the highest alpha coefficient as the final version of Dependability for the 56-item CPAI.

Using the same procedure, we got the final version of Accommodation and Interpersonal Relatedness. The alpha coefficients of the 3 combinations of Accommodation were from 0.676 to 0.768, with an average of 0.712. As for Interpersonal Relatedness, there were 1,458 combinations (3*4*3*3*3*3). The alpha coefficients for these combinations were from 0.644 to 0.724, with an average of 0.681. It is worth noting that the two authors later thought that an item in harmony scale was inappropriate in content, so only three items were left to select from. The combinations of items and their alpha coefficients are available on the Open Science Framework at https://mfr.osf.io/render?url=https%3A%2F%2Fosf.io%2F2pev3%2Fdownload.

Thus, we got the highest alpha coefficients of each domain and the final version of the 56-item CPAI. The alpha coefficients are 0.822 for Social Potency, 0.831 for Dependability, 0.768 for Accommodation, and 0.724 for Interpersonal Relatedness, with an average of 0.786. The item-total correlations of each item with the domain to which it belongs range from 0.271 to 0.649, with only one below 0.40 and an average of 0.522.

We also conducted Velicer’s minimum average partial correlation procedure to determine the number of components of the 56 items. When the fourth component was extracted, the average squared partial correlation reached a minimum value of 0.0031, a result that supports a four-factor solution (Velicer, 1976).

Then, we conducted ESEM to test the four-factor model of the 56-item CPAI. These analyses included models with and without CUs. As shown in Table 2, only the ESEM model with CUs provides an adequate fit. Table 3 demonstrates the standardized factor loadings, item-total correlations, and factor correlations. Factor loadings tend to be modest. Target loadings of the ESEM model range from 0.15 to 0.615, with six loadings below 0.30 and a median of 0.408. Cross-loadings in the ESEM model range from −0.323 to 0.366. Almost 80 percent of them (134 out of 168) are statistically different from zero. Five cross-loadings are higher than 0.30, and five items have cross-loading higher than the target loading. R-squares of items range from 0.097 to 0.386, with six below 0.160 and a median of 0.253. Factor correlations range from −0.396 to 0.140, with a median of −0.028.

**Table 2:** Summary of Goodness of Fit Statistics for ESEM Models.

Model	*χ* ^2^	*df*	RMSEA (90%CI)	TLI	CFI
56-item CPAI	17101.38	1,322	0.033 (0.032; 0.033)	0.854	0.829
56 with CUs	9721.41	1,293	0.024 (0.024; 0.025)	0.907	0.922
28-item CPAI	2439.82	272	0.027 (0.026; 0.028)	0.921	0.943

*N=11,137; CFI, Comparative fit index; TLI, Tucker-Lewis Index; RMSEA, Root Mean Square Error of Approximation; CI, Confidence Interval; CUs, a priori correlated uniquenesses based on the scale design of the CPAI-2; 56 with CUs, 56-item CPAI with CUs; all χ^2^ values are significant at p<0.01*.

**Table 3:** Means, Item-Total correlations, Standardized Factor Loadings, R-squares, and Factor Correlations of the ESEM models with CUs.

items	Means	Item-Total	ESEM				
			SP	De	Ac	IR	R-square
413	3.669	0.468	**0.382**	−0.117	−0.021	0.183	0.218
159	2.881	0.442	**0.325**	0.161	−0.013	−0.096	0.134
82	3.357	0.611	**0.568**	−0.019	−0.034	−0.003	0.325
308	3.266	0.530	**0.487**	−0.081	−0.168	0.003	0.265
431	3.372	0.593	**0.565**	−0.146	−0.165	−0.013	0.354
243	3.579	0.506	**0.441**	0.112	0.162	0.089	0.234
325	2.996	0.586	**0.535**	0.007	−0.106	−0.110	0.299
373	3.216	0.616	**0.579**	−0.092	−0.119	−0.043	0.351
53	3.548	0.587	**0.544**	0.032	0.055	0.106	0.324
246	3.658	0.540	**0.488**	−0.044	0.048	0.211	0.322
196	2.880	0.474	**0.346**	0.087	−0.020	−0.022	0.125
315	3.379	0.536	**0.480**	0.096	0.083	0.059	0.246
55	2.692	0.471	**0.498**	0.261	0.025	−0.323	0.383
3.468	0.498	**0.421**	0.006	0.016	0.191	0.235
184	3.213	0.477	**0.410**	−0.058	−0.114	−0.001	0.181
135	3.614	0.476	**0.393**	−0.008	0.009	0.290	0.270
539	2.906	0.527	−0.129	**0.479**	−0.055	0.135	0.271
434	2.780	0.556	−0.125	**0.475**	−0.085	0.054	0.279
521	2.706	0.539	−0.003	**0.380**	−0.258	−0.037	0.296
28	3.075	0.448	0.051	**0.390**	0.049	0.051	0.139
17	3.243	0.428	−0.062	**0.368**	−0.084	0.229	0.195
216	3.351	0.271	0.185	**0.150**	−0.108	0.147	0.097
353	2.646	0.406	−0.031	**0.182**	−0.281	−0.034	0.160
170	2.782	0.549	0.013	**0.483**	−0.063	−0.057	0.272
331	2.537	0.547	0.037	**0.414**	−0.148	−0.105	0.267
351	2.978	0.524	−0.088	**0.486**	0.049	0.110	0.229
375	2.562	0.593	0.044	**0.615**	0.073	−0.105	0.372
183	2.966	0.471	−0.092	**0.397**	−0.075	0.123	0.196
5	2.784	0.516	0.081	**0.399**	−0.095	−0.050	0.210
117	2.637	0.578	0.098	**0.468**	−0.096	−0.143	0.307
269	2.090	0.508	0.005	**0.288**	−0.302	−0.202	0.315
11	2.555	0.577	0.004	**0.512**	−0.031	−0.130	0.310
388	2.853	0.589	−0.047	**0.588**	0.021	0.064	0.335
147	3.187	0.506	−0.029	**0.524**	0.047	0.185	0.270
274	3.234	0.549	0.029	−0.193	**0.406**	−0.084	0.258
419	3.212	0.565	0.013	−0.142	**0.388**	−0.040	0.210
34	3.638	0.538	−0.004	−0.278	**0.245**	0.102	0.216
371	3.533	0.640	0.007	−0.128	**0.554**	0.027	0.386
327	3.701	0.569	−0.075	−0.311	**0.234**	0.276	0.324
380	2.843	0.490	−0.119	−0.106	**0.321**	−0.141	0.162
217	3.029	0.492	0.021	−0.082	**0.418**	−0.233	0.230
321	3.255	0.564	0.057	−0.250	**0.345**	−0.045	0.248
489	3.746	0.649	−0.055	−0.303	**0.348**	0.219	0.381
144	3.512	0.634	0.042	−0.285	**0.339**	0.094	0.300
484	3.582	0.456	0.075	0.027	−0.136	**0.304**	0.112
99	4.079	0.546	0.171	−0.101	0.028	**0.450**	0.281
370	4.052	0.534	0.155	0.009	0.015	**0.439**	0.235
129	3.687	0.507	0.008	−0.071	−0.232	**0.374**	0.169
278	3.800	0.497	0.284	−0.069	0.033	**0.368**	0.262
286	3.744	0.454	0.174	0.141	0.113	**0.353**	0.187
236	3.538	0.484	0.096	−0.084	−0.199	**0.372**	0.178
364	3.422	0.493	−0.074	0.037	−0.227	**0.463**	0.237
215	3.538	0.466	0.366	0.053	0.024	**0.291**	0.244
445	3.523	0.502	0.016	−0.024	−0.205	**0.340**	0.138
394	3.452	0.526	0.004	−0.038	−0.298	**0.384**	0.201
158	4.042	0.549	0.170	0.057	0.138	**0.526**	0.357
**Factor correlation**							
	SP		1.0				
	De		−0.045^*^	1.0			
	Ac		−0.001	−0.396^**^	1.0		
	IR		0.128^**^	−0.127^**^	0.140^**^	1.0	

*N=11,137; Item-Total, Item-Total correlation SP, Social Potency; De, Dependability; Ac, Accommodation; IR, Interpersonal Relatedness; All item-total correlations are significant at p<0.01. The number of each item in this table is its original numbers in CPAI-2*.

* *p<0.05*;

** *p<0.01*.

### Developing the 28-Item CPAI

We created a 28-item CPAI by dropping one of the two items selected from each of the 28 personality scales. The two criteria for deleting items were both based on the ESEM solution of the 56-item CPAI. That is, items with low factor loading or worse cross-loading problems would be dropped. Worse cross-loading problems included more cross-loadings on one item and the absolute values of cross-loading higher than or closer to that of the target loading. Thus, we got the 28-item CPAI.

For this even shorter version of CPAI-2, the alpha coefficients are 0.710 for Social Potency, 0.760 for Dependability, 0.609 for Accommodation, and 0.590 for Interpersonal Relatedness, with an average of 0.667. The item-total correlations of each item with the domain to which it belongs are from 0.509 to 0.671, with an average of 0.584 (See Table 4). The ratio of the mean alpha reliability is 0.849 for the 28-item CPAI compared to the 56-item CPAI. In addition, we regarded the 28-item CPAI as a part of the 56-item CPAI and computed the part-whole correlations for each domain. The part-whole correlations are 0.939 for Social Potency, 0.940 for Dependability, 0.919 for Accommodation, and 0.900 for Interpersonal Relatedness, with an average of 0.925. The mean of the squared part-whole correlations is 0.855. These results suggest that the 28-item CPAI is about 15% less reliable than the 56-item CPAI.

**Table 4:** Means, Item-Total correlations, Standardized Factor Loadings, R-squares, and Factor Correlations of the ESEM model of the 28-item CPAI.

items	Means	Item-Total	ESEM				
			SP	De	Ac	IR	R-square
82	3.357	0.624	**0.539**	0.010	−0.003	0.024	0.293
308	3.266	0.583	**0.510**	−0.022	−0.100	0.003	0.272
431	3.372	0.642	**0.631**	−0.065	−0.070	−0.036	0.407
373	3.216	0.635	**0.610**	−0.020	−0.033	−0.032	0.372
196	2.880	0.509	**0.322**	0.140	0.034	0.039	0.115
315	3.379	0.557	**0.417**	0.103	0.084	0.111	0.198
524	3.468	0.538	**0.398**	0.051	0.055	0.222	0.228
184	3.213	0.526	**0.436**	−0.009	−0.051	0.016	0.195
434	2.780	0.588	−0.116	**0.447**	−0.135	0.060	0.281
521	2.706	0.560	0.009	**0.344**	−0.293	−0.031	0.295
170	2.782	0.595	0.020	**0.482**	−0.069	−0.051	0.276
331	2.537	0.588	0.056	**0.454**	−0.104	−0.081	0.276
351	2.978	0.556	−0.076	**0.493**	0.039	0.130	0.235
375	2.562	0.651	0.046	**0.660**	0.099	−0.063	0.404
183	2.966	0.511	−0.091	**0.396**	−0.069	0.138	0.193
117	2.637	0.591	0.076	**0.463**	−0.091	−0.086	0.279
388	2.853	0.622	−0.058	**0.580**	0.012	0.099	0.329
419	3.212	0.588	−0.018	−0.153	**0.365**	−0.034	0.197
34	3.638	0.560	−0.020	−0.264	**0.266**	0.102	0.227
371	3.533	0.671	−0.031	−0.101	**0.573**	0.050	0.400
217	3.029	0.570	0.002	−0.081	**0.420**	−0.219	0.221
144	3.512	0.630	0.017	−0.284	**0.337**	0.089	0.302
99	4.079	0.577	0.146	−0.114	0.006	**0.444**	0.269
129	3.687	0.587	0.001	−0.084	−0.268	**0.398**	0.194
278	3.800	0.545	0.289	−0.034	0.065	**0.381**	0.277
364	3.422	0.578	−0.070	0.031	−0.249	**0.472**	0.243
394	3.452	0.580	0.012	−0.072	−0.334	**0.394**	0.218
158	4.042	0.583	0.145	0.032	0.089	**0.516**	0.321
**Factor correlation**							
	SP		1.0				
	De		−0.092^**^	1.0			
	Ac		−0.010	−0.413^**^	1.0		
	IR		0.120^**^	−0.178^**^	0.174^**^	1.0	

*N=11,137; Item-Total, Item-Total SP, Social Potency; De, Dependability; Ac, Accommodation; IR, Interpersonal Relatedness; all item-total correlations are significant at p<0.01; the number of each item in this table is its original numbers in CPAI-2*.

^*^p<0.05;

** *p<0.01*.

As shown in Table 2, the fit of the ESEM model is acceptable. Table 4 demonstrates the standardized factor loadings, item-total correlations, and factor correlations of the ESEM model. Factor loadings are modest. Specifically, the target loadings of the ESEM model range from 0.266 to 0.660, with only one loading less than 0.30 and a median of 0.446. The cross-loadings in the ESEM model range from −0.334 to 0.289. More than 80% of them (70 out of 84) are statistically different from zero. Only one cross-loading is higher than 0.30, and none of the items has a cross-loading higher than the target loading. Factor correlations range from −0.413 to 0.174 with a median of −0.051.

### Measurement Invariance Across Gender

We conducted multiple-group ESEM to test the measurement invariance of the 56-item CPAI and the 28-item CPAI across gender. We first tested the 13 models of the 28-item CPAI and labeled them with the letter A in Table 5. As for the 56-item CPAI, we tested two sets of the 13 models, one in which the CUs were allowed to vary for females and males and another in which the CUs were constrained to be invariant over responses by females and males. We labeled the former with the letter B and the latter with the letter C in Table 5. In general, we conducted three sets of measurement invariance tests: set A, set B and set C.

**Table 5:** Summary of Goodness of Fit Statistics for All Gender Invariance Models.

Model	*χ* ^2^	*df*	TLI	CFI	NFParm	RMSEA (90%CI)
**Model 1−No invariance (Configural Invariance)**						
Model 1A	2733.40	544	0.919	0.942	324	0.027 (0.026; 0.028)
Model 1B	11235.82	2,586	0.904	0.920	718	0.025 (0.024; 0.025)
Model 1C	11268.87	2,615	0.905	0.920	689	0.024 (0.024; 0.025)
**Model 2: FL−Weak factorial/measurement IN (Nested with Model 1)**						
Model 2A	2971.31	640	0.928	0.939	228	0.026 (0.025; 0.027)
Model 2B	11654.93	2,794	0.909	0.918	510	0.024 (0.024; 0.024)
Model 2C	11683.57	2,823	0.910	0.918	481	0.025 (0.025; 0.026)
**Model 3: FL and Uniq (Nested with Model 1, Model 2)**						
Model 3A	3062.77	668	0.929	0.937	200	0.025 (0.025; 0.026)
Model 3B	11826.18	2,850	0.910	0.917	454	0.024 (0.023; 0.024)
Model 3C	11855.29	2,879	0.911	0.917	425	0.024 (0.023; 0.024)
**Model 4: FL+FVFC (Nested with Model 1, Model 2)**						
Model 4A	3023.76	650	0.928	0.938	218	0.026 (0.025; 0.027)
Model 4B	11713.59	2,804	0.909	0.917	500	0.024 (0.024; 0.024)
Model 4C	11743.22	2,833	0.910	0.917	471	0.024 (0.023; 0.024)
**Model 5: FL+Int−Strong factorial/measurement invariance (Nested with Model 1, Model 2)**						
Model 5A	3182.84	664	0.925	0.934	204	0.026 (0.025; 0.027)
Model 5B	12116.13	2,846	0.907	0.914	458	0.024 (0.024; 0.025)
Model 5C	12144.10	2,875	0.908	0.914	429	0.024 (0.024; 0.025)
**Model 6: FL+FVCV+Uniq (Nested with Model 1–4)**						
Model 6A	3117.69	678	0.929	0.936	190	0.026 (0.025; 0.026)
Model 6B	11885.20	2,860	0.910	0.916	444	0.024 (0.023; 0.024)
Model 6C	11915.08	2,889	0.911	0.916	415	0.024 (0.023; 0.024)
**Model 7: FL+Int+Uniq−strict factorial/measurement invariance (Nested with Model 1–3, 5)**						
Model 7A	3276.13	692	0.926	0.932	176	0.026 (0.025; 0.027)
Model 7B	12289.85	2,902	0.908	0.913	402	0.024 (0.024; 0.025)
Model 7C	12317.95	2,931	0.909	0.913	373	0.024 (0.024; 0.025)
**Model 8: FL+FVCV+Int (Nested with Model 1, 2,4, 5)**						
Model 8A	3237.57	674	0.925	0.933	194	0.026 (0.025; 0.027)
Model 8B	12177.41	2,856	0.907	0.914	448	0.024 (0.024; 0.025)
Model 8C	12206.25	2,885	0.908	0.914	419	0.024 (0.024; 0.025)
**Model 9: FL+FVCV+Int+Uniq (Nested with Model 1–8)**						
Model 9A	3333.25	702	0.926	0.931	166	0.026 (0.025; 0.027)
Model 9B	12351.33	2,912	0.907	0.912	392	0.024 (0.024; 0.025)
Model 9C	12380.18	2,941	0.908	0.912	363	0.024 (0.024; 0.025)
**Model 10: FL+Int+FMn−latent mean IN (Nested with Model 1, 2, 5)**						
Model 10A	3438.79	668	0.918	0.928	200	0.027 (0.027; 0.028)
Model 10B	12363.30	2,850	0.905	0.912	454	0.025 (0.024; 0.025)
Model 10C	12391.12	2,879	0.905	0.912	425	0.024 (0.024; 0.025)
**Model 11: FL+Int+FMn+Uniq−manifest mean IN (Nested with Model 1–3, 5, 7, 10)**						
Model 11A	3533.39	696	0.919	0.926	172	0.027 (0.026; 0.028)
Model 11B	12537.34	2,906	0.905	0.911	398	0.024 (0.024; 0.025)
Model 11C	12565.11	2,935	0.906	0.911	369	0.024 (0.024; 0.025)
**Model 12: FL+FVCV+Int+FMn (Nested with Model 1, 2, 4–6, 8, 10)**						
Model 12A	3492.10	678	0.918	0.926	190	0.027 (0.027; 0.028)
Model 12B	12422.95	2,860	0.905	0.911	444	0.025 (0.024; 0.025)
Model 12C	12451.73	2,889	0.905	0.911	415	0.024 (0.024; 0.025)
**Model 13: FL+FVCV+Int+FMn+Uniq−complete factorial IN (Nested with Model 1–12)**						
Model 13A	3589.33	706	0.919	0.925	162	0.027 (0.026; 0.028)
Model 13B	12597.43	2,916	0.905	0.910	388	0.025 (0.024; 0.025)
Model 13C	12625.87	2,945	0.906	0.910	359	0.024 (0.024; 0.025)

*Women n=5,565; Men n=5,464; χ2=chi-square statistic; df, degrees of freedom; CFI, Comparative fit index; TLI, Tucker-Lewis Index; NFParm, number of free parameters; RMSEA, Root Mean Square Error of Approximation; CI, Confidence Interval; CUs, a priori correlated uniquenesses based on previous works; FL, factor loadings; Uniq, item uniquenesses (error variance); FVCV, factor variances-covariances; Int, item intercepts; FMn, factor means. All χ^2^ values are significant at p<0.01*.

#### Configural Invariance

The goodness of fit statistics provides adequate support for the configural invariance models (Model 1A, Model 1B, and Model 1C), with all of the TLI and CFI exceeding 0.90 and all of the RMSEA below 0.05.

#### Weak Invariance

When factor loadings were constrained to be equal across gender, the TLIs and the RMSEAs are even better than those in Model 1, except for the RMSEA in Model 2C. None of the ΔCFIs exceeds 0.01, with ΔCFIs of 0.003 in model 2A and 0.002 in both models 2B and 2C. The ΔRMSEAs does not exceed 0.015 in model 2A, 2B, and 2C. The results support weak invariance among the three sets of the test across gender.

#### Strong Invariance

The strong invariance models constrain both factor loading and item intercepts to be equal across gender. The fit statistics do not reject invariant intercepts hypothesis, with the ΔCFIs below 0.01 and the ΔRMSEAs below 0.015 in model 3A, 3B, and 3C.

#### Strict Invariance

The strict invariance models require equal factor loadings, item intercepts, and uniquenesses across gender. When compared with models 5A, 5B, and 5C, the corresponding models 7A, 7B, and 7C do not produce substantial changes in TLI, CFI, and RMSEA. We also compared all the other various pairs of models (Model 3 vs. Model 2; Model 6 vs. Model 4; Model 9 vs. Model 8; Model 11 vs. Model 10; Model 13 vs. Model 12) to test the invariance of the uniquenesses and yielded the same results. These results provide good support for the strict measurement invariance for the three sets of the test.

#### CUs Invariance

We compared each Model B with corresponding Model C to examine whether the CU invariance across gender could be established. All ΔCFIs do not change except for the one (0.001) in the comparison between Model 12C and Model 12B. All ΔRMSEAs are below 0.015, and all TLI increase by 0.001, except for one of the comparison between Model 10C and Model 10B does not change. The results support invariance of CUs.

#### Factor Variance\Covariance Invariance

We compared several pairs of models, including Model 4 vs. Model 2, Model 6 vs. Model 3, Model 8 vs. Model 5, Model 9 vs. Model 7, and Model 12 vs. Model 10, and Model 13 vs. Model 11, with all ΔCFIs less than 0.002, all ΔRMSEAs less than 0.001 and all TLIs unchanged in all sets of the test. The results suggest that the factor variance\covariance is invariant between males and females.

#### Factor Mean Invariance

We tested factor mean invariance across gender by comparing four pairs of models: M10 vs. M5, M11 vs. M7, M12 vs. M8, and M13 vs. M9. What these four models (M10-M13) have in common is that they all have factor means constrained to zero for both male and female groups. The results show that all changes in model fit indices do not exceed the cut-points to reject the invariant factor means hypothesis. However, in the test of set A for the 28-item CPAI, the differences in fit indices only marginally support invariance. Changes in both CFI and TLI exceed 0.005, with ΔCFIs equaling to 0.006 and changes in TLI equaling to 0.007.

We could explain gender differences in terms of latent means with sufficient justification since there had been reasonable support for the strict invariance over gender. Thus, we examined models in which means were constrained to 0 for the male group and freely estimated for the female group. It was apparent that females yielded significantly higher scores on Dependability, Accommodation, and Interpersonal Relatedness and lower scores on Social Potency. Table 6 presents a summary of the standardized gender differences based on the four models that provided estimates of these differences.

**Table 6:** Summary of gender differences on latent mean factors.

Models	56-item CPAI (set B)				28-item CPAI (set A)			
	SP	De	Ac	IR	SP	De	Ac	IR
Model 5	−0.195	0.056	0.219	0.258	−0.219	0.090	0.190	0.300
Model 7	−0.194	0.056	0.218	0.258	−0.218	0.090	0.190	0.302
Model 8	−0.188	0.102	0.174	0.235	−0.222	0.123	0.215	0.198
Model 9	−0.188	0.097	0.178	0.230	−0.222	0.123	0.215	0.198

*Women n=5,565; Men n=5,464; SP, Social Potency; De, Dependability; Ac, Accommodation; IR, Interpersonal Relatedness; all difference values are significant at p<0.01*.

We also performed independent sample t tests to examine gender differences in the four factors of both short forms and found the same pattern as the multi-group ESEM results. Females scored higher than males on Dependability, Accommodation, and Interpersonal Relatedness, but lower on Social Potency. However, except for differences in social competence, the effect sizes for most gender differences are very small and of little practical significance. These results can explain why the factor mean invariance could be established in multi-group ESEM analyses. Table 7 demonstrated a summary of the t test statistics.

**Table 7:** Summary of the *t* test statistics for gender differences of the four factors of both short forms.

Factors		56-item CPAI				28-item CPAI			
		means	*SD*	.	Cohen’s *d*	means	*SD*	.	Cohen’s *d*
SP	Male	3.35	0.58	8.268	0.17	3.32	0.64	8.809	0.15
	Female	3.25	0.60			3.22	0.66		
De	Male	2.79	0.64	−3.886	0.06	2.73	0.74	−3.359	0.07
	Female	2.83	0.61			2.78	0.71		
Ac	Male	3.34	0.67	−5.872	0.11	3.36	0.74	−4.077	0.07
	Female	3.41	0.66			3.41	0.72		
IR	Male	3.68	0.54	−5.613	0.09	3.71	0.61	−7.446	0.13
	Female	3.73	0.52			3.79	0.59		

*Women n=5,565; Men n=5,464; SP, Social Potency; De, Dependability; Ac, Accommodation; IR, Interpersonal Relatedness; SD, Standardized Deviation; all t values are significant at p<0.01*.

### Criterion Validity

We tested the correlations between the four domains of both short forms and several criterion variables, including the big five factors and several health-related variables. As shown in Table 8, the pattern of criterion associations is very similar across the two short forms. We calculated the correlations between the two columns of the criterion associations for each domain of the CPAI. We found correlations of 1.000 for Social Potency, 1.000 for Dependability, 0.999 for Accommodation, and 0.993 for Interpersonal Relatedness, suggesting that the 28-item CPAI is almost identical with the 56-item CPAI in terms of the relationship between the domains and the criterion variables.

**Table 8:** Correlations between the four factors of the two short forms with other variables.

	28-item CPAI				56-item CPAI			
	SP	De	Ac	IR	SP	De	Ac	IR
Extraversion	0.506^**^	0.299^**^	0.207^**^	0.029	0.519^**^	0.312^**^	0.243^**^	0.095
Agreeableness	0.081	0.472^**^	0.435^**^	0.334^**^	0.079	0.476^**^	0.467^**^	0.311^**^
Conscientiousness	0.344^**^	0.585^**^	0.337^**^	0.428^**^	0.341^**^	0.583^**^	0.379^**^	0.432^**^
Emotional Stability	0.345^**^	0.680^**^	0.439^**^	0.290^**^	0.345^**^	0.670^**^	0.469^**^	0.291^**^
Openness	0.662^**^	0.292^**^	0.128^*^	0.109^*^	0.668^**^	0.306^**^	0.164^**^	0.138^*^
PHQ	−0.312^**^	−0.555^**^	−0.294^**^	−0.068	−0.319^**^	−0.555^**^	−0.365^**^	−0.074
GAD	−0.246^**^	−0.565^**^	−0.327^**^	−0.132^*^	−0.255^**^	−0.554^**^	−0.399^**^	−0.136^*^
GHQ	−0.454^**^	−0.607^**^	−0.374^**^	−0.214^**^	−0.457^**^	−0.599^**^	−0.435^**^	−0.237^**^
Social dysfunction	−0.528^**^	−0.540^**^	−0.315^**^	−0.297^**^	−0.532^**^	−0.522^**^	−0.370^**^	−0.326^**^
Anxiety	−0.223^**^	−0.507^**^	−0.349^**^	−0.043	−0.225^**^	−0.506^**^	−0.403^**^	−0.048
Loss of confidence	−0.307^**^	−0.468^**^	−0.266^**^	−0.131^*^	−0.307^**^	−0.478^**^	−0.311^**^	−0.150^**^
SWB	0.363^**^	0.390^**^	0.270^**^	0.168^**^	0.384^**^	0.381^**^	0.299^**^	0.209^**^

*N=330. SP, Social Potency, De, Dependability, Ac, Accommodation, IR, Interpersonal Relatedness, PHQ, Patient Health Questionnaire, GAD, Generalized Anxiety Disorder, GHQ, General Health Questionnaire. SWB, Subjective Well-Being*.

* *p<0.05*;

** *p<0.01*.

The four domains of both short forms are significantly correlated with almost all big five factors. Specifically, Social Potency has stronger correlations with Extraversion (.=0.506) and Openness (.=0.662) than with other big five factors. Dependability has stronger correlations with Conscientiousness (.=0.585) and Emotional Stability (.=0.680). Accommodation has stronger correlations with Agreeableness (.=0.435) and Emotional Stability (.=0.439). IR has relatively weak correlations with the big five factors, comparing with the other three domains. Social Potency does not correlate with Agreeableness and IR does not correlate with Extraversion.

As for health-related variables, the four domains are significantly correlated with PHQ (ranging from −0.294 to −0.555), GAD (ranging from −0.246 to −0.565), GHQ (ranging from −0.374 to −0.607), social dysfunction (ranging from −0.315 to −0.540), anxiety (ranging from −0.223 to −0.507), loss of confidence (ranging from −0.266 to −0.478), and subjective well-being (ranging from 0.270 to 0.390). Among them, Dependability has relatively strong correlations with PHQ, GAD, GHQ, Social dysfunction and Anxiety, Social Potency has relatively strong correlation with Social dysfunction, and IR is not correlated with PHQ and anxiety.

## Discussion

The CPAI-2 is a promising instrument in the fields of personality psychology and cross-cultural psychology. However, shortages of short forms may slow down its progress in these fields. In the present study, we developed two short forms with sound psychometric qualities for the CPAI-2: the 56-item CPAI and the 28-item CPAI. Then, we examined the extent to which these short forms retain the structure of the CPAI-2 and their measurement invariance across gender. It turns out that they both share the same four-factor structure of the CPAI-2, and the four factors appear to be distinct from each other. Both short forms demonstrate strict invariance across gender. Further tests show that men scored higher than women on social competence. In addition, both short forms have adequate reliabilities and validities.

In the present study, we provided alpha coefficients for each domain of the two short forms and examined the relationship between CPAI domains and several criterion variables. Among the four domains, Accommodation and Interpersonal Relatedness are the two domains with relatively low internal consistency in both short forms. In the 56-item CPAI, the alpha coefficients of the four domains are all higher than 0.7, indicating adequate internal consistency (Nunnally and Bernstein, 1994). When reducing the number of items by half to construct the 28-item CPAI, the alpha coefficients decreased in all four domains, with lower internal consistency in two of them, dropping below 0.70. That is to say, in the 28-item CPAI, Accommodation and Interpersonal Relatedness seems to be weak in internal consistency, especially Interpersonal Relatedness.

The way we constructed the short forms prioritizes high bandwidth over high internal consistency. We selected the same number of items from each of the 28 CPAI-2 scales so that each domain of the short forms would cover all of its aspects in the original CPAI-2, a strategy that resulted in a relatively high level of item content heterogeneity in each domain. Item content heterogeneity refers to whether the items in a scale cover many different aspects of one trait or focus on only a few (McCrae et al., 2011). The high item content heterogeneity can lead to low internal consistency. For example, Interpersonal Relatedness consists of six diverse aspects. In the 56-item CPAI, there are two items per aspect, whereas in the 28-item CPAI, there is only one item per aspect. Thus, the former is less heterogeneous because it has a peer that reflects the same aspect in each item. Interpersonal Relatedness is weaker than other domains in terms of internal consistency, probably also because the aspects that make it up are more heterogeneous in terms of content.

We placed more emphasis on validity than on internal consistency reliability. Low internal consistency caused by item content heterogeneity may not lead to low validity (McCrae et al., 2011). In terms of validity, the 28-item CPAI does not appear to be worse than the 56-item CPAI according to the correlation pattern between the four domains and those criterion variables. Specifically, the four domains of both short forms are positively correlated with subjective well-being and negatively correlated with variables indicating poor mental health, and Dependability seemed to be the most potent protector of health among them.

In addition, domains of short forms are widely related to the big five personality traits. The way they correlated with the big five traits is quite similar to the way the scales of CPAI domains are entangled with the facets of the big five factors in previous joint factor analyses (Cheung et al., 2001, 2008). For example, Scales of Dependability mainly combined with facets of Neuroticism (Emotional Stability) and Conscientiousness in previous joint factor analyses of CPAI measures and big five measures. Then, in the present study, the Dependability of both short forms was apparently more strongly correlated with Emotional Stability and Conscientiousness. The short forms are in excellent consistency with the original CPAI measures regarding their relationship with the Big Five personality factors.

In addition, the correlation pattern of CPAI domains and the big five factors provides evidence of convergent and discriminant validity from a multi-trait-multi-method perspective. The big five and CPAI measures are developed with different approaches, the former uses an etic approach, while the latter uses a combined etic-emic approach. However, the personality traits they measured overlap. Dependability overlaps with Emotional Stability and Conscientiousness, Social potency overlaps with Openness and Extraversion, and Accommodation overlaps with agreeableness. These overlaps are reflected in previous joint factor analyses and are again demonstrated in these correlations in present study. The correlations between one CPAI domain and the big five factors overlapping with it are much higher than those between the domain and other CPAI domains.

The short forms do offer substantial savings in assessment time compared to the full CPAI-2. According to Soto and John (2017a), the 60-item BFI-2 takes 4 to 10min to complete, and the 30-item BFI-2-S takes 3 to 5min. The 56-item CPAI and the 28-item CPAI have about the same number of items as BFI-2 and BFI-2-S, respectively. Thus, we can infer from their estimates of the time required to complete the 56-item CPAI (4 to 10min) and the 28-item CPAI (3 to 5min). When using the short form of the CPAI-2, the time would shrink from half an hour to less than 10min, a decrease that would allow more time for other variables or substantially reduce the likelihood of fatigue and impatience. This is why short forms are preferred over the full version, especially when they have comparable reliability and validity.

However, the efficiency gains in short forms often come at the cost of reliability and validity (Soto and John, 2017a), meaning that short forms need larger samples to maintain the same statistic power as the full CPAI-2. The cost of short forms also includes weakening or even losing hierarchies. The full CPAI-2 is appropriate for both domain-level and scale-level personality assessment, a hierarchical assessment that combines the benefits of high bandwidth with high fidelity (Soto and John, 2017b). The 56-item CPAI retains to some extent the capability to assess personality hierarchically and is only appropriate for scale-level assessment in very large samples. The 28-item CPAI, however, lacks the capacity to assess scale-level personality traits.

Thus, it is easy to choose between the CPAI-2 and the 56-item CPAI, but not between the 56-item CPAI and the 28-item CPAI. Compared to the CPAI-2, the 56-item CPAI allows a time advantage of more than 20min, but with a slight attenuation in psychometric qualities and the capacity of hierarchical measurement. It seems to be worth it. However, it would not be worthwhile to replace the 56-item CPAI with the 28-item CPAI to save less than 7min at the cost of weakened reliability and loss of hierarchical measurement ability. As advised by Soto and John (2017a), the 28-item CPAI is suitable for studies in which assessment time and respondent fatigue are core concerns, and even small gains in efficiency are critical.

We conducted multi-group ESEM analyses to test the measurement invariance of the two short forms in a comprehensive taxonomy of invariance models with appropriate tests of full measurement and structural invariance. The results support configural invariance across gender and invariance of factor loadings, item intercepts and uniquenesses, correlated uniquenesses, factor variances and covariances, and factor means for both short forms. At the level of measurement invariance, strict gender invariance has been established which implies that the two short instruments are comparable between men and women in the structural level, including factor variance and covariance, and factor mean.

The invariance of the factor covariance indicates that the correlation pattern among the four factors is the same between males and females. Thus, we can expect the short forms will have the same discriminant and convergent validity when applied to different gender groups. Factor mean invariance across gender indicates that there is no gender difference in the four factors. However, the results of the t test show significant gender differences with small effect sizes. These two results are not really contradictory because most of the effect sizes of gender differences are too small to be of any practical significance, except for the gender differences in social competence. Men scored higher than women on social competence, with a small but not negligible effect size, a result that is consistent with the findings on scale-level gender differences on the personality traits of the CPAI-2 (Cheung et al., 2004). Cheung et al. (2004) also found that males scored higher than females on some scales of dependability, while females scored higher than males on other scales of dependability. Such scale-level differences offset each other on domain-level, explaining why gender difference is trivial and negligible on dependability.

Previous studies on the structure of the CPAI used traditional EFA approaches that could only provide a crude comparison across groups (Cheung et al., 2003; Lin and Church, 2004). Lin and Church (2004) conducted the CFA to test the structure of CPAI scales and NEO-FFI facets and found the CFA model did not fit the data well. Thus, we believe that the best option currently available for performing measurement invariance analysis for CPAI instruments is the ESEM models. We have now provided a basis for cross-sex comparisons of the short forms of CPAI through the ESEM models. In the future, we will use these models for research that compare personality traits of CPAI across different cultures.

## Conclusion

The work reported here provides two short forms for CPAI-2. Both of them are time efficient, gender invariant, and have adequate validity. One has 56 items and the other 28 items. The former retains a certain degree of the capacity of hierarchical measurement, and the latter is more time-saving. Henceforth, we have the flexibility to choose different versions of CPAI depending on the study. In addition, the present study provides new evidence for the advantages of ESEM and reveals its potential applicability in future studies on CPAI.

## Data Availability Statement

The raw data supporting the conclusions of this article will be made available by the authors, without undue reservation.

## Ethics Statement

This study was carried out in accordance with the recommendations of the Ethics guidelines of the Ethics Committee of Institute of Psychology, Chinese Academy of Sciences. The study protocol was approved by the institutional review board: Ethics Committee of Institute of Psychology, Chinese Academy of Sciences (reference number: H20020).

## Author Contributions

FC, JZ, MZ, and FR had the initial ideas. JZ, MZ, FC, and FL collected the data. MZ, DH, and FL analyzed the data. MZ and DH wrote the drafts and the final manuscript. JZ, WF, and WM reviewed the several drafts of the manuscript. MZ and DH revised the manuscript. All authors approved the final version of the manuscript.

## Funding

This work was funded by the National Natural Science Foundation of China (grant no. 71774156) granted to MZ.

## Conflict of Interest

The authors declare that the research was conducted in the absence of any commercial or financial relationships that could be construed as a potential conflict of interest.

## Publisher’s Note

All claims expressed in this article are solely those of the authors and do not necessarily represent those of their affiliated organizations, or those of the publisher, the editors and the reviewers. Any product that may be evaluated in this article, or claim that may be made by its manufacturer, is not guaranteed or endorsed by the publisher.
